# Infection by *Mycoplasma* spp., feline immunodeficiency virus and feline leukemia virus in cats from an area endemic for visceral leishmaniasis

**DOI:** 10.1186/s13071-018-2716-9

**Published:** 2018-03-20

**Authors:** Mary Marcondes, Karina Y. Hirata, Juliana P. Vides, Ludmila S. V. Sobrinho, Jaqueline S. Azevedo, Thállitha S. W. J. Vieira, Rafael F. C. Vieira

**Affiliations:** 1 São Paulo State University (Unesp), School of Veterinary Medicine, Araçatuba, São Paulo, Brazil; 2 Department of Veterinary Medicine, Universidade Federal do Paraná, Curitiba, Paraná, Brazil

**Keywords:** *Leishmania infantum*, *Ehrlichia* spp., *Babesia* spp., *Anaplasma platys*, PCR

## Abstract

**Background:**

Visceral leishmaniasis (VL) has been increasingly recognized in cats living in areas endemic for the disease. Co-infection with *Leishmania infantum* and other infectious agents is well established in dogs. However, for cats, data on co-infections with *L. infantum* and other infectious agents are still sparse. The aim of this study was to identify the prevalence of vector-borne pathogens, *Mycoplasma* spp., feline immunodeficiency virus (FIV) and feline leukaemia virus (FeLV) in cats from an area endemic for VL in southeastern Brazil.

**Results:**

Of the 90 cats, eight (8.9%) were infected with *Mycoplasma* spp., five (5.5%) were FIV- positive and one (1.1%) was FeLV-positive. Co-infection with *L. infantum* and at least one other infectious agent was found in 9/50 (18.0%; CI: 8.6–31.4%) cats. In Group 1 (cats infected naturally by *L. infantum*), 4/50 (8.0%) cats were positive for FIV, 4/50 (8%) for *Mycoplasma* spp. and 1/50 (2.0%) was co-infected with FeLV and *Mycoplasma* spp. In Group 2 (cats non-infected with *L. infantum*), 2/40 (5.0%) cats were infected with *Mycoplasma* spp. and 1/40 (2.5%) was co-infected with FIV and *Mycoplasma* spp. All cats were negative for *Ehrlichia* spp., *Babesia* spp. and *Anaplasma platys.*

**Conclusion:**

A low prevalence of co-infection in *Leishmania*-infected and non-infected cats was found. Co-infections with *Leishmania* and vector-borne diseases in cats are not common in this area endemic for VL in Brazil.

## Background

Visceral leishmaniasis (VL) is a neglected zoonotic disease caused by *Leishmania infantum*, with dogs acting as the main reservoir for this protozoon pathogen. Visceral leishmaniasis has been recognized increasingly in cats living in areas endemic for the disease [1–4]. While infected cats are less frequently ill than dogs, probably due to a natural feline resistance, clinical disease has been associated with immunosuppressive co-infections with feline immunodeficiency virus (FIV) and feline leukemia virus (FeLV) [5].

Although cats are exposed to arthropod parasites, their lifestyle habits may be a limiting factor for transmission of arthropod-borne pathogens, particularly those transmitted by ticks, compared with dogs [6]. In some regions of Brazil, cats are infested by ticks, such as *Rhipicephalus sanguineus* (sensu *lato*) [7–10], and thus may be infected with the pathogens they may transmit. Previous studies have found Brazilian free-roaming cats infected with *Mycoplasma* spp. and feline vector-borne pathogens (FVBPs) such as species of *Babesia*, *Anaplasma* or *Ehrlichia* [11, 12].

In areas endemic for VL, co-infections of *L. infantum* and other infectious agents are common in dogs [13–17]. Co-infection may potentiate disease pathogenesis and alter clinical manifestations, complicating diagnosis and treatment, and influencing prognosis [18]. Recent studies have been performed worldwide to characterize co-infections with *L. infantum* and other infectious agents, including other vector-borne pathogens (VBP), in cats [19–22]. In Brazil, data on co-infections are still sparse and limited to reports of co-infections with the major feline retroviruses, FIV and FeLV [19], *Toxoplasma gondii* [19, 23, 24] and *Neospora caninum* [24]. Therefore, the aim of this study was to identify co-infections with FVBPs, *Mycoplasma* spp., FIV and FeLV in *Leishmania-*infected and non-infected cats from an area endemic for VL in southeastern Brazil.

## Methods

### Animals

Ninety mixed-breed cats of different ages and sex, either presented to a veterinary teaching hospital (VTH) (*n* = 38 cats) or living at two cat shelters (*n* = 52 cats), in an area endemic for VL in Araçatuba, São Paulo, southeastern Brazil, were sampled between March 2014 and May 2015. Signalment and clinical data recorded included gender, age (sometimes estimated by assessment of dentition) and clinical signs reported by the cat owner or the person in charge of the cat shelter. A thorough physical examination was performed on each cat by a registered veterinary surgeon. The two shelters were sampled because they were known to have a high prevalence of *L. infantum* infection. According to the *Leishmania* infection status, based on blood and/or bone marrow polymerase chain reaction (PCR) testing, cats were subdivided into two groups: Group 1 (G1) included 50 cats infected naturally with *L. infantum*, and Group 2 (G2) included 40 cats known not to be infected with *L. infantum*. Cats were eligible for the study if: (i) they were at least 6 months-old; (ii) they had not been diagnosed or treated for leishmaniasis in the past; (iii) they had not received drugs with known anti-*Leishmania* activity for the previous 6 months; and (iv) they had not received immune-modulating drugs for the previous 3 months.

### Sampling

Blood samples and bone marrow aspirates were collected by a registered veterinary surgeon. Blood samples were collected by jugular venipuncture. One ml was placed into citrate tubes (BD Vacutainer®, Becton Dickinson, Franklin Lakes, NJ, USA) for PCR analysis (*Leishmania* spp., *Ehrlichia* spp., *Anaplasma platys*, *Mycoplasma* spp*.* and *Babesia* spp.) and kept at -80 °C until testing. Three milliliters were placed into tubes without anticoagulant and kept at room temperature (25 °C) until visible clot retraction; the samples were then centrifuged at 1500×*g* for 5 min, serum separated and kept at -20 °C for serological studies. Bone marrow aspirates were collected for *Leishmania* PCR analysis from the iliac crest or femur of cats under anesthesia with a combination of ketamine (5 mg/kg body weight; Quetamina®, Vetnil, São Paulo, Brazil) and midazolam (0.3 mg/kg body weight; Dormonid®, Roche, Rio de Janeiro, Brazil). Samples were aspirated into heparinized syringes and then gently expelled into a sterile glass petri dish. Spicules were collected from the dish into sterile glass capillary tubes and then into citrate tubes (BD Vacutainer®, Becton Dickinson, Franklin Lakes, NJ, USA) tubes for PCR analysis, and kept at -80 °C until analyzed. Concurrently, one drop of the spicules collected from each sample was placed onto a glass microscope slide and a squash preparation was made. The slide was stained by Diff-Quick (Panótico Rápido®, Laborclin, São Paulo, Brazil) and evaluated cytologically by a veterinary clinical pathologist to confirm the presence and quality of bone marrow in the sample.

### Serological testing

All serum samples were tested for FeLV p27 antigen and IgG antibodies against FIV, by a commercial enzyme-linked immunosorbent assay (ELISA), rapid assay kit (SNAP® FIV Antibody/FeLV Antigen Combo Test: IDEXX Laboratories, Westbrook, ME, USA).

### DNA extraction

After thawing at room temperature, 200 μl of whole blood and bone marrow were subjected to DNA extraction using a commercial kit (QIAamp™ DNA Mini Kit Blood and Tissue, Qiagen, Valencia, CA, USA), according to the manufacturer’s instructions. Negative control purifications using ultrapure water were performed in parallel to monitor cross-contamination in each batch of 30 samples. The concentration and purity of extracted DNA were assessed by spectrophotometry (ND-1000, Nano-Drop Technologies, Wilmington, DE, USA) by measuring the absorbance at 260 and 280 nm, respectively. Thereafter, DNA aliquots were stored at -20 °C until molecular testing.

### Detection of *Leishmania* DNA by real-time polymerase chain reaction

The target *Leishmania* DNA for PCR amplification was a 116 base pair (bp) fragment in the constant region of the kinetoplast DNA minicircle, using primers described previously [25]. Briefly, the reaction was performed using a commercial mastermix with SYBR Green fluorophore (SYBRGreen JumpStart TaqReadMix S4438, Sigma-Aldrich, St Louis, MO, USA), 900 nM of each primer and 5 μl of DNA, in a final volume of 25 μl. Samples from blood and bone marrow (tested in triplicate) were placed into 96-well PCR plates and PCR amplification was carried out in a thermocycler (CFX96TM Real-Time System, Bio-Rad, Hercules, CA, USA) using the following conditions: 94 °C for 2 min, 40 cycles of 94 °C for 15 s, followed by 60 °C for 1 min, when fluorescence data were collected. To carry out melting curve analysis, the temperature was increased from 60 °C to 95 °C, with an increment of 0.5 °C every 5 s. The cycle threshold (Ct) value was calculated for each sample by determining the point at which the fluorescence exceeded the threshold limit. Each amplification run contained a positive control (DNA extracted from 1.6 × 10^4^ *L. infantum* promastigotes) in triplicate, to test the proper conditions of the reagents, and negative controls with ultrapure water in triplicate to monitor cross-contamination.

### Detection of *Ehrlichia* spp., *Anaplasma platys*, *Mycoplasma* spp. and *Babesia* spp. by conventional polymerase chain reaction

A conventional PCR for the housekeeping gene glyceraldehyde-3-phosphate dehydrogenase (GAPDH) was performed to ensure successful DNA extraction, as previously described [26]. Samples were evaluated using conventional PCR with genus-specific primers targeting a portion of the *16S* rDNA gene of *Ehrlichia* spp. (344 bp) [27], *A. platys* (359 bp) [28] and *Mycoplasma* spp. (*c.*900 bp) [29, 30], and a portion of the *18S* rDNA gene of *Babesia* spp. (*c*.500 bp) [31]. For each PCR assay, DNA from dogs known to be infected with *Ehrlichia* spp., *A. platys* and *Babesia* spp., and cats known to be infected with *Mycoplasma* spp., and nuclease-free water were used as positive and negative controls, respectively. The amplified PCR products were subjected to gel electrophoresis in 1.5% agarose gels for 1 h at 100 V, followed by SYBR safe staining (6 μg/ml; SYBR® Safe DNA Gel Stain, Invitrogen, CA, USA), and were viewed under a 312 nm UV light transilluminator.

### Sequencing

Amplicons obtained randomly from eight *Leishmania*-positive and all eight *Mycoplasma* spp.-positive samples were purified, evaluated by spectrophotometry for concentration and purity (Nanodrop™ 2000 Spectrophotometer, Thermo Fisher Scientific, Wilmington, MA, USA), and sequenced in both directions by the Sanger method. Thereafter, sequences were subjected to BLASTn analysis [32] for determining the identity with the sequences deposited in the GenBank database. Sequencing was restricted to these 16 samples due to financial constraints.

### Statistical analysis

Chi-square test was used to determine if age and sex were associated with infections, if *Leishmania* infection was associated with FIV, FeLV and *Mycoplasma* infection status and if FIV and *Mycoplasma* infections were associated. Fisher’s exact test was used to determine if there was association between FeLV and *Mycoplasma*, and FIV and FeLV infections. Odds ratios (OR), 95% confidence intervals and *P*-values were calculated, and results were considered significant when *P* < 0.05. Data were compiled and analyzed by Epi Info™ Software (version 7.1.5, CDC).

## Results

A total of 28/90 (31.1%) male and 62/90 (68.9%) female cats, all of mixed-breed, with ages ranging from 0.5 to 10 years (median 2 years), were included in the study. Among the *Leishmania*-infected cats (G1), 40/50 (80.0%) were living in one of the shelters and 10/50 (20.0%) were referred to the VTH, 17/50 (34.0%) were male and 33/50 (66.0%) female, with ages ranging from 0.5 to 10 years (median 2 years). In G1, 20/50 (40.0%, 95% CI: 26.4–54.8%) cats had evidence of clinical abnormalities on physical examination, including alopecia-hypotrichosis (*n* = 9; 45.0%), weight loss (*n* = 7; 35.0%), lymph node enlargement (*n* = 5; 25.0%), ulcerative skin lesions (*n* = 4; 20.0%), dehydration (*n* = 3; 15.0%), conjunctivitis (*n* = 2; 10.0%), mucosal pallor (*n* = 1; 5.0%), uveitis (*n* = 1; 5.0%), mucopurulent nasal discharge (*n* = 1; 5.0%), sneezing (*n* = 1; 5.0%), stomatitis (*n* = 1; 5.0%), vomiting (*n* = 1; 5.0%), diarrhea (*n* = 1; 5.0%) and jaundice (*n* = 1; 5.0%). Among the non-infected cats (G2), 12/40 (30.0%) were living in one of the shelters and 28/40 (70.0%) were referred to the VTH, 11/40 (27.5%) were male and 29/50 (72.5%) female, with ages ranging from 0.5 to 10 years (median 2.5 years). In G2, 12/40 (30.0%, 95% CI: 16.6–46.5%) cats showed clinical signs including lymph node enlargement (*n* = 6; 50.0%), weight loss (*n* = 4; 33.3%), ulcerative skin lesions (*n* = 3; 25.0%), ocular and/or nasal discharge (*n* = 3; 25.0%), depression (*n* = 1; 8.3%), inappetence (*n* = 1; 8.3%), mucosal pallor (*n* = 1; 8.3%), oral mucosal ulceration (*n* = 1; 8.3%), conjunctival hyperaemia (*n* = 1; 8.3%), and haematuria (*n* = 1; 8.3%),while the other 28/40 (70.0%, 95% CI: 53.5–83.4%) were healthy and had been referred to the VTH for neutering.

Cytological evaluation confirmed that all samples were of bone marrow, and in seven *Leishmania*-infected cats (14.0%), all presenting with clinical signs, *Leishmania* spp. amastigotes were observed in bone marrow cytology. The GAPDH gene was consistently amplified from all samples subjected to PCR. In 58.0% (*n* = 29) of the infected cats *Leishmania* DNA was amplified from both bone marrow and blood. In 28.0% (*n* = 14) and 14.0% (*n* = 7) of the cats, *Leishmania* DNA was amplified only from bone marrow and blood, respectively. Of the 90 cats, eight (8.9%, 95% CI: 3.9–16.7%) were infected with *Mycoplasma* spp., five (5.5%, 95% CI: 1.8–12.5%) were FIV-positive and one (1.1%, 95% CI: 0.03–6.0%) was FeLV-positive. Co-infection with *Leishmania* and at least one other infectious agent was found in 9/50 (18.0%, 95% CI: 8.6–31.4%) cats. In G1 4/50 (8.0%, 95% CI: 2.2–19.2%) cats were positive for FIV, 4/50 (8.0%, 95% CI: 2.2–19.2%) for *Mycoplasma* spp. and 1/50 (2.0%, 95% CI: 0.05–10.6%) was co-infected with FeLV and *Mycoplasma* spp. The later animal was a 4 year-old male cat co-infected with FeLV and “*Candidatus* Mycoplasma haemominutum”. The cat was referred to the VTH with weight loss, pale mucous membranes with packed cell volume (PCV) of 20%, dehydration, sneezing and serous mucopurulent nasal discharge, which led to a suspicion of feline herpesvirus-1 (FHV-1) infection; an ulcerative lesion at the base of the left pinna suggesting a neoplastic process, that was further not confirmed, and flea infestation. The cat did not return for the next consultation since it disappeared from the house on the next day after the visit to the hospital.

In G2, 2/40 (5.0%, 95% CI: 0.6–16.9%) cats were infected with *Mycoplasma* spp. and 1/40 (2.5%, 95% CI: 0.06–13.2%) was co-infected with FIV and *Mycoplasma* spp. All cats were negative for *Ehrlichia* spp., *Babesia* spp. and *A. platys.*

Association between sex and infection by *Leishmania* (*χ*^2^ = 0.4381, *df* = 1, *P* = 0.5081), FIV (*χ*^2^ = 2.0615, *df* = 1, *P* = 0.1511) and FeLV (*χ*^2^ = 2.2392, *df* = 1, *P* = 0.1346) was not observed. Male cats were more likely to be infected with *Mycoplasma* spp. (*χ*^2^ = 7.8916, *df* = 1, *P* = 0.0050). Association between positivity for *Leishmania* and FIV (*χ*^2^ = 1.2812, *df* = 1, *P* = 0.2577), *Leishmania* and FeLV (*χ*^2^ = 0.8090, *df* = 1, *P* = 0.3684), *Leishmania* and *Mycoplasma* spp. (*χ*^2^ = 0.1715, *df* = 1, *P* = 0.6788), FIV and FeLV (*P* = 1.0000, OR: unable to calculate), FIV and *Mycoplasma* spp. (*χ*^2^ = 0.8070, *df* = 1, *P* = 0.3690), and FeLV and *Mycoplasma* spp. (*P* = 0.0889, OR: undefined) was not observed. The prevalence of infectious pathogens in *Leishmania-*infected and non-infected cats for each variable evaluated is summarized in Table 1.

**Table 1:** Prevalence of infectious pathogens in *Leishmania*-infected and non-infected cats from an area endemic for visceral leishmaniasis in Brazil

	FIV		FeLV		*Mycoplasma* spp.	
	+/*n* (%)	*P*-value	+/*n* (%)	*P*-value	+/*n* (%)	*P*-value
Cats infected with *Leishmania* (G1)						
Sex						
Male	2/18 (11.1)	0.6123	1/18 (5.0)	0.3600	4/18 (22.2)	0.0500
Female	2/32 (6.2)		0/32 (0)		1/32 (3.1)	
Age						
> 1 year	4/32 (12.5)	0.1178	1/32 (3.0)	1.000	5/32 (15.6)	0.0770
≤ 1 year	0/18 (0)		0/18 (0)		0/18 (0)	
Cats non-infected with *Leishmania* (G2)						
Sex						
Male	1/10 (10.0)	0.2500	0/10 (0)	na	2/10 (20.0)	0.1480
Female	0/30 (0)		0/30 (0)		1/30 (3.3)	
Age						
> 1 year	1/20 (5.0)	1.000	0/20 (0)	na	3/20 (15.0)	0.2307
≤ 1 year	0/20 (0)		0/20 (0)		0/20 (0)	

*Abbreviations: FIV* feline immunodeficiency vírus, *FeLV* feline leukaemia virus, *+* number of positive cats, *n* total number, *na* not applicable

Nucleotide sequences from eight *Leishmania*-infected cats had ≥ 99% identity with multiple *L. infantum* kDNA gene sequences deposited in GenBank (KJ417491, AB678348, EU437407, EU437406, EU437405). Four out of the eight *Mycoplasma*-positive samples sequenced showed ≥ 98% identity with multiple “*Ca*. M. haemominutum” *16S* rDNA gene sequences deposited in GenBank (accession nos. KU852585, EU839983, AY150981), two and one sequences showed ≥ 99% identity with *Mycoplasma haemofelis* (accession nos. KM 275241, KM275239), and “*Candidatus* Mycoplasma turicensis” (accession no. KM275263), respectively. Multiple attempts to amplify the *16S* rDNA from the remaining *Mycoplasma*-positive sample were unsuccessful.

In G1, two cats were co-infected with “*Ca*. M. haemominutum”, two with *Mycoplasma haemofelis* and one with “*Ca*. M. turicensis”. In G2, two cats were infected with “*Ca*. M. haemominutum” and in one cat *Mycoplasma* DNA sequencing was unsuccessful.

## Discussion

Feline vector-borne diseases (FVBDs) have been less investigated than canine vector-borne diseases (CVBDs) in part because of difficulties in making a diagnosis of FVBD since there are fewer commercially available diagnostic tests. Additionally, the research community that focuses on FVBDs is smaller than that which studies CVBDs. Finally, it is suggested that diseases in cats are diagnosed less frequently, since cats are not taken for veterinary consultation as often as dogs [6]. Even with the introduction of molecular techniques, since prevalence data for FVBDs are scarce, veterinarians sometimes may not consider the possibility of occurrence of those diseases [33]. Although some studies have investigated FVBD prevalence among cat populations worldwide [20–22, 34–37], a limited number of such studies have been reported from Brazil [11, 12, 36, 38], except for investigations of feline leishmaniasis [4, 19, 24, 39–43].

The present study is the first to evaluate co-infections in *Leishmania*-infected and non-infected cats from an area endemic for VL in Brazil. In the same area, a previous study in dogs reported a *Leishmania* seroprevalence of 48.0% [44]. Additionally, dogs from the same endemic area for VL have been shown to be co-infected with *Leishmania* and *E. canis*, *B. vogeli*, *T. gondii* [14] and *N. caninum* [44].

The cat population studied herein was a convenience sample. Sampling was performed on animals attending a VTH or residing in a local cat shelter environment. However, the cat population provided a good spectrum of healthy and clinically ill animals with two different lifestyles.

In order to increase the sensitivity of detection of *Leishmania* DNA, both blood and bone marrow samples were collected. PCR on bone marrow has been reported to be more sensitive than using whole blood for the diagnosis of canine leishmaniasis [45]. In a previous study, *Leishmania* DNA was amplified from bone marrow, whole blood and in both samples from 22.0%, 22.0% and 7.32% of the infected cats, respectively [46]. Conversely, in the present study, bone marrow was the more sensitive sample for the diagnosis of infection (*n* = 43 cats; 86.0%) compared with blood (*n* = 36 cats; 72.0%). If only whole blood had been used to detect *Leishmania* DNA, 10.0% of the cats would have had a false-negative PCR result.

In the present study, 18.0% of *Leishmania*-infected cats were co-infected with at least one infectious agent. Although *Leishmania* co-infection is commonly related to infection with FIV [19, 47, 48] and FeLV [48, 49], the cats studied herein were also co-infected with feline hemoplasmas, as previously observed in cats from Cyprus [21].

The prevalence of FIV infection in the present study was 5.5%. Co-infection with  *Leishmania* and FIV was observed in 8.0% cats, while 2.5% of the non-infected cats were FIV-positive. Three of the *Leishmania-*FIV co-infected cats had skin lesions similar to those previously observed by Vides et al. [4] in cats with VL. In general, the animals were in good body condition, without evidence of other systemic disease or clinical signs of FIV infection. In the same area as the present study, a previous study has found an association between co-infection with *Leishmania* spp. and FIV, suggesting that cats living in areas endemic for VL are significantly more likely to be co-infected with FIV [19].

Previous studies have found an association between *Leishmania* and FeLV infection [47]. The low number of FeLV-infected cats evaluated in the present study has impaired statistical analysis. A previous study in the same area endemic for VL reported a FeLV prevalence of 0.33%, although co-infection with *Leishmania* and FeLV was not observed [19]. The only cat co-infected with *Leishmania* and FeLV in the present study, was also infected with “*Ca*. M. haemominutum”. The cat was referred to the VTH due to an upper respiratory tract infection, probably caused by FHV-1 and feline calicivirus (FCV), associated with FeLV infection [50]. The ulcerative lesion on the pinna was similar to those observed previously in cats with VL [4]. While hematological abnormalities in cats infected with “*Ca*. M. haemominutum” may be minor or absent, co-infection with “*Ca*. M. haemominutum” and FeLV can cause severe anaemia [51], which could explain the low PCV observed in the cat. Although there are few reports of cats co-infected with *Leishmania* and two or more infectious agents [19, 52], to the authors’ best knowledge the present study represents the first report of a cat co-infected with *Leishmania*, FeLV and “*Ca*. M. haemominutum”.

Hemoplasma infection is a relatively common finding in cats worldwide [21, 53–55], and these infections are widely recognized in Brazil [11, 56–58]. In the present study, 8.9% of the cats were positive for *Mycoplasma* spp., with “*Ca*. M. haemominutum” being the most prevalent species (50.0%), similar to previous studies [11, 21, 58–60]. It is important to note that a hemoplasma screening method was used herein, and therefore, co-infections by multiple hemoplasma species may have been missed. Global prevalence data on feline hemoplasmas range from 6.5% to 38.5% [11, 53–55, 57, 58, 60]. Differences in hemoplasma prevalence may be attributed to the sensitivity of the assay (i.e. conventional PCR vs quantitative PCR), the population studied, the geographical location and sampling methods (i.e. convenience non-randomized samples vs non-convenience randomized samples).

Impaired immunocompetence (e.g. caused by immunosuppressive FIV and/or FeLV co-infection) has been associated historically with enhanced pathogenicity of *Mycoplasma* spp. [54, 55, 61]. In Brazil, a previous study has found an association between co-infection with “*Ca*. M. haemominutum” and FIV [62]. Herein, besides the cat that was co-infected with *Leishmania*, FeLV and “*Ca*. M. haemominutum”, one cat was co-infected with FIV and “*Ca*. M. haemominutum”, one with *Leishmania* and “*Ca*. M. haemominutum”, and one single infected with “*Ca*. M. haemominutum”. Except for the first, the other three cats had a red blood cell (RBC) count within the normal range (data not shown). Despite most infections with “*Ca*. M. haemominutum” being chronic and not associated with anaemia [63, 64], previous studies have reported cats with haemolytic anaemia where no apparent causative agent other than “*Ca*. M. haemominutum” was identified, although primary immune-mediated haemolytic anaemia may have been the underlying cause in some or all of those cats [64]. To the best of the authors’ knowledge, this is the first study documenting co-infection with *L. infantum* and *Mycoplasma* spp. in cats from South America. Further studies should be conducted to better elucidate the association between *L. infantum* and feline hemoplasmas.

Recent studies have focused on providing data on FVBDs [20, 22, 34, 35, 37]. A few have also evaluated *Mycoplasma* spp. infection [21, 37] with *L. infantum*-infected cats being seven times more likely to be infected with “*Ca*. M. turiscensis” [21]. Feline hemoplasma infection has been associated previously with male sex [64], in agreement with the present study, where male cats were eight times more likely to be infected with *Mycoplasma* spp. (*P* = 0.0050).

All of the present cats were negative for the tick-borne pathogens (TBPs) species *Ehrlichia* and *Babesia*, similar to previous studies [22]. There are many hypotheses to explain why cats may be less prone to arthropod-borne diseases, including their grooming behavior that could remove ticks before pathogen transmission, and a natural, genetically controlled immunological resistance to arthropods and the microorganisms they may transmit [6].

Previous studies in cats have reported low prevalence rates for some TBPs [20, 36, 37]. Molecular detection followed by sequencing studies in Brazilian cats revealed a low prevalence of infection by *E. canis* [65, 66], *A. platys* [67] and closely related species [12, 68, 69]. In contrast, a significant molecular prevalence of *Babesia* spp. infection was found in Brazilian cats [11, 12], in agreement with previous studies on cats in Portugal [20]. *Rhipicephalus sanguineus* (*s.l.*) ticks, the main vector of these TBPs, are widely distributed in Brazil [70]. Although reports of infestation by this tick species in cats are rare [7–10], *R. sanguineus* (*s.l.*), has been described as parasitizing cats [71]. In the present study, none of the animals were infested by ticks at the time of clinical examination, which may have contributed to the TBP negative status.

## Conclusions

A low prevalence of co-infection with FVBDs in either *Leishmania*-infected and non-infected cats was found in this study. Infection with TBPs are not common in cats in this area endemic for VL in Brazil. To our knowledge, this is the first study documenting co-infection with *L. infantum* and *Mycoplasma* spp. in cats from South America.

## Abbreviations

CVBD: canine vector-borne diseases
ELISA: enzyme-linked immunosorbent assay
FCV: feline calicivirus
FeLV: feline leukemia virus
FHV-1: feline herpesvirus-1
FIV: feline immunodeficiency virus
FVBD: feline vector-borne diseases
FVBP: feline vector-borne pathogens
GAPDH: glyceraldehyde-3-phosphate dehydrogenase
OR: odds ratio
PCR: polymerase chain reaction
PCV: packed cell volume
RBCs: red blood cells
TBP: tick-borne pathogen
VBP: vector-borne pathogens
VL: visceral leishmaniasis
VTH: veterinary teaching hospital
